# Long‐term survival of dogs treated for gallbladder mucocele by cholecystectomy, medical management, or both

**DOI:** 10.1111/jvim.15611

**Published:** 2019-09-06

**Authors:** Max Parkanzky, Janet Grimes, Chad Schmiedt, Scott Secrest, Andrew Bugbee

**Affiliations:** ^1^ Department of Small Animal Medicine and Surgery University of Georgia College of Veterinary Medicine Athens Georgia; ^2^ Department of Veterinary Biosciences and Diagnostic Imaging University of Georgia College of Veterinary Medicine Athens Georgia

**Keywords:** abdominal ultrasound, biliary rupture, biliary sludge, ursodiol

## Abstract

**Background:**

Gallbladder mucoceles (GBM) typically are treated by cholecystectomy. Medical management rarely has been reported and medical and surgical management have not been compared.

**Hypothesis/Objectives:**

To compare survival of dogs treated for GBM by medical management or cholecystectomy or both.

**Animals:**

Eighty‐nine client‐owned dogs diagnosed with GBM that received cholecystectomy or medical treatment or both from 2011 to 2017.

**Methods:**

Potential cases were identified by searching the medical records database. Data collected included signalment, clinicopathologic results, treatments, and ultrasonographic images and reports. Dogs were grouped according to the treatment received (medical management, surgical treatment, or both) that was chosen at the discretion of the attending veterinarian. Survival analysis was performed and prognostic variables identified and compared between treatment groups.

**Results:**

Of dogs surviving at least 14 days after diagnosis, median survival times were 1802 (95% confidence interval [CI], 855‐not reached) days, 1340 (95% CI, 444‐1340) days, and 203 (95% CI, 18‐525) days, for the surgical, medical, and medical then surgical treatment groups, respectively, and differed significantly (*P* < .0001). Gallbladder mucocele type (*P* = .05), serum alkaline phosphatase activity (*P* = .0001), and serum creatinine (*P* = .002) and phosphorus (*P* = .04) concentrations were associated with decreased survival across groups. Suspicion of biliary rupture on abdominal ultrasound (AUS) examination was correlated with increased survival in the surgical group (*P* = .02).

**Conclusions and clinical importance:**

Cholecystectomy for the treatment of GBM results in the best long‐term survival in dogs surviving the immediate postoperative period (14 days) compared to medical management. Although medical management is associated with shorter survival compared to surgical treatment, it is a reasonable alternative when surgery cannot be pursued.

**Abbreviations**
ALP: alkaline phosphatase
AUS: abdominal ultrasound
CI: confidence interval
GBM: gallbladder mucocele
Med: medical treatment group
Med‐Sx: medical then surgical treatment group
Sx: surgical treatment group
UDCA: ursodeoxycholic acid

## 1 INTRODUCTION

Gallbladder mucoceles (GBM) are characterized by the accumulation of tenacious mucin‐laden bile in the gallbladder, which can result in cholestasis and affect gallbladder wall integrity.^1^, ^2^ Historically, GBM have been associated with mortality rates of 22 to 40% in the immediate postoperative period.^1^, ^2^, ^3^, ^4^, ^5^, ^6^, ^7^, ^8^ More recent studies have reported improved mortality rates in patients surviving the immediate postoperative period, but data from cases in the past 15 years are infrequently reported.^7^, ^9^, ^10^ Older dogs of a variety of breeds can be affected by GBM, with Shetland Sheepdogs,^1^, ^6^, ^11^ Miniature Schnauzers,^11^, ^12^ and Cocker Spaniels^1^, ^5^, ^8^, ^11^, ^12^ being over‐represented in the literature.

The etiopathogenesis of GBM in dogs is unclear and likely to be multifactorial. Several reports have described an increased prevalence of underlying endocrinopathies such as hyperadrenocorticism, hypothyroidism, and hyperlipidemia in patients with GBM.^11^, ^13^, ^14^, ^15^ Studies using an experimental model of hyperadrenocorticism have failed to identify a cause and effect relationship between iatrogenic hypercortisolism and GBM.^16^, ^17^ Additional research investigating the administration of various drugs^18^ and the role of genetic mutations^19^, ^20^ similarly have been unable to define a specific etiology for GBM formation. Compelling evidence supports the theory that gallbladder dysmotility, decreased bile flow or stasis, and altered bile composition contribute to the formation of mucoceles but whether these abnormalities represent discrete diseases or secondary manifestations of other systemic diseases is unknown.^21^, ^22^, ^23^

Although surgical intervention typically has been described for the treatment of GBM, medical management also has been reported.^2^, ^24^ Historically, most patients with GBM are presented for acute signs associated with extrahepatic biliary obstruction or rupture of the gall bladder such as jaundice, vomiting, lethargy, polyuria, polydipsia, diarrhea, and abdominal pain.^4^ Little is known about the early stages of the disease including clinical presentation, ultrasonographic appearance of early GBM, or if earlier intervention with either surgical or medical management may alter the prognosis or course of the disease. Several recent reports describe the clinical course of biliary sediment^25^, ^26^, ^27^ and early GBM,^12^ but no study describes response of biliary sediment or early GBM to medical treatment. The purpose of our study was to investigate the outcomes associated with medical versus surgical management of GBM and to identify clinical, clinicopathologic, and diagnostic imaging variables associated with prognosis and survival.

## 2 MATERIALS AND METHODS

### 2.1 Animals

Electronic medical records were retrospectively searched for dogs that were diagnosed with GBM from 2011 to 2017. Records were searched for the keywords “gallbladder,” “mucocele,” and “gallbladder mucocele.” Dogs were included if a diagnosis of GBM was suspected upon ultrasound examination and was treated medically, surgically by cholecystectomy, or both. Decisions regarding treatment were made by the attending veterinarian upon consultation with clients and were not standardized because of the retrospective nature of the study. Dogs were excluded if no medical or surgical treatments were pursued, non‐GBM biliary disease (eg, cholelithiasis, non‐GBM biliary obstruction, cholangitis, non‐GBM cholecystitis) or nonbiliary mucocele (salivary mucocele) were diagnosed, the patient was euthanized at presentation, or biliary diversion surgery was performed.

### 2.2 Data collection

Data collected from medical records included signalment (age, sex, reproductive status, and breed), medical, or surgical treatments performed (cholecystectomy or medical treatment with ursodeoxycholic acid [UDCA] with or without other medications or supplements), and results of clinicopathologic testing (CBC and serum biochemistry). Ultrasound images of the gallbladder were retrospectively reviewed by a single board‐certified radiologist (S.S.) who was blinded to treatment and outcome and scored according to previously described criteria.^12^ Briefly, images of the gallbladder were classified into the following GBM types: type 1, immobile echogenic bile; type 2, incomplete stellate pattern; type 3, typical stellate pattern; type 4, kiwi‐like pattern and stellate combination; type 5, kiwi‐like pattern with residual central echogenic bile; and type 6, kiwi‐like pattern. Suspicion for gallbladder rupture based on available ultrasound reports was recorded. Criteria for suspicion of biliary rupture were not standardized because of the retrospective nature of the study and our desire to more accurately represent factors that may have influenced clinical decision making for each case, but pericholecystic effusion and focal‐to‐diffuse hyperechoic mesentery and loss of continuity of the gallbladder wall were considered suspicious of biliary rupture. Dogs then were grouped together based on whether patients underwent cholecystectomy (surgical treatment [Sx group]), received medical treatment (Med group), or failed medical management and subsequently were treated by surgery (Med‐Sx group). Referring veterinarians were contacted for additional follow‐up data (ie, survival status and date of death, if applicable).

### 2.3 Statistical analysis

Survival time was calculated as the time between the date of diagnosis and death. Patients were censored at the time of analysis if they were alive or lost to follow‐up. Kaplan‐Meier analysis with Cox proportional hazards regression was performed to assess differences in survival among treatment groups. The survival curves for medical management and surgery crossed, indicating that the assumption of proportional hazards between treatment groups was not met. Therefore, survival analysis was repeated for patients that survived or were not censored before 14 days after initial diagnosis to account for postoperative mortality.

Both 2‐group (Sx versus Med and Med‐Sx combined) and 3‐group (Sx versus Med versus Med‐Sx) analyses were performed to accurately represent all cases where medical management was pursued. Two‐group analyses consisted of Fisher's exact tests used to compare sex and breed, and Student's *t* test to compare age and GBM type between 2 treatment groups. In the 3‐group model, log‐rank tests were used to compare survival among treatment groups, sexes, and breeds after 14 days from initial diagnosis. Cox proportional hazards analyses were used to test for effects of age and ultrasonographic GBM type on survival and to calculate hazard ratios (HRs) for treatment groups, before and after 14 days since diagnosis, for age, sex, breed, and GBM type.

Twenty‐eight variables (Table 1) were identified among clinicopathologic data and imaging findings as potential prognostic factors for survival and were tested for interaction using multivariate Cox proportional hazards models. Serum total bilirubin concentration also was dichotomized (≤0.3 versus >0.3) based on prognostic findings of previous studies.^4^, ^5^, ^9^ Each multivariable Cox proportional hazards model included factors for treatment group (medically managed, surgery, medically managed, and then surgery) and each potential prognostic factor 1 at a time and an interaction of treatment group and potential prognostic factor. The treatment group and prognostic factor interaction term tests if the relationship between the prognostic factor and survival was consistent across treatment groups. If the treatment group and prognostic factor interaction effect had *P* < .15, then HRs were calculated for each treatment group separately. If the treatment group and prognostic factor interaction effect had *P* > .15, then a univariable Cox proportional hazards model was run for each prognostic factor separately and HRs were calculated for all treatment groups together.

**Table 1:** Ultrasound variables and clinicopathologic analytes versus survival

Variable	Reference	*P* value^a^	Group	Mean ± SD	Hazard ratio
GBM type	NA	.05*	All	NA	1.41 (1.01‐1.98)*
Suspected gallbladder rupture in ultrasound report	NA	.02^b^	Med	NA	0.86 (0.11‐6.9)
			Med‐Sx	NA	1.66 (0.33‐8.38)
			Sx	NA	0.21 (0.06‐0.76)*
WBC (×10^3^ cells/μL)	4.2‐12.9	.39	All	16.5 ± 9.6	1.02 (0.98‐1.05)
Hct (%)	42.2‐59.8	.25	All	42.4 ± 7.36	0.97 (0.93‐1.05)
Segs (×10^3^ cells/μL)	2.7‐8.5	.37^b^	Med	11.33 ± 8.0	1.08 (0.99‐1.18)
			Med‐Sx	13.07 ± 6.56	0.94 (0.84‐1.05)
			Sx	16.16 ± 9.42	1.02 (0.96‐1.07)
Bands (×10^3^ cells/μL)	0‐0.3	.41	All	0.32 ± 0.74	0.75 (0.35‐1.62)
Lymphs (×10^3^ cells/μL)	0.5‐4.1	.12	All	1.17 ± 0.75	0.59 (0.29‐1.18)
Monos (×10^3^ cells/μL)	0.1‐1	.72	All	1.17 ± 1.21	1.05 (0.49‐1.4)
Eos (×10^3^ cells/μL)	0‐1.2	.96	All	0.22 ± 0.33	0.98 (0.35‐2.76)
Platelets (×10^3^ cells/μL)	226‐424	.16	All	337.1 ± 159.3	1.002 (0.999‐1.004)
Sodium (mmol/L)	143‐150	.18	All	146 ± 5.18	1.06 (0.97‐1.17)
Potassium (mmol/L)	3.5‐5.1	.99	All	4.61 ± 0.65	1.002 (0.53‐1.91)
Chloride (mmol/L)	106‐114	.34	All	108 ± 13.3	0.99 (0.97‐1.01)
Bicarbonate (mmol/L)	16‐25	.60	All	20‐3.6	0.96 (0.84‐1.11)
BUN (mg/dL)	9‐28	.25	All	18.9 ± 16.1	1.01 (0.99‐1.03)
Creatinine (mg/dL)	0.6‐1.3	.002*	All	0.96 ± 0.65	2.63 (1.54‐4.47)*
Total protein (g/dL)	5.4‐7.1	.82	All	5.95 ± 0.96	1.04 (0.72‐1.52)
Albumin (g/dL)	3.3‐4.2	.08	All	3.2 ± 0.7	0.6 (0.34‐1.05)
ALP (U/L)	13‐95	<.0001*^,^ b	Med	1544 ± 2250	1.02 (0.99‐1.05)^c^
			Med‐Sx	2564 ± 3596	0.98 (0.96‐1.002)^c^
			Sx	2847 ± 2987	1.05 (1.02‐1.07)*^,^ c
ALT (U/L)	12‐106	.94	All	610 ± 744	1.00002 (0.9995‐1.0005)
Glucose (mg/dL)	76‐120	.28	All	102 ± 41.9	1.004 (0.998‐1.01)
Calcium, ionized (mmol/L)	1.2‐1.5	.99	All	1.22 ± 0.16	1.01 (0.08‐12.31)
Calcium, total (mg/dL)	9.5‐11.2	.34	All	10.23 ± 1.092	1.18 (0.84‐1.67)
Phosphorus (mg/dL)	2.7‐5.2	.04*	All	4.3 ± 1.87	1.26 (1.05‐1.51)*
Magnesium (mg/dL)	1.8‐2.4	.71	All	2.17 ± 0.33	0.75 (0.17‐3.38)
Cholesterol (mg/dL)	109‐345	.53	All	372 ± 224	1.001 (0.999‐1.002)
Bilirubin (mg/dL)	0‐0.3	.60	All	3.1 ± 5.1	1.02 (0.96‐1.08)
Bilirubin ≤0.3 versus >0.3	NA	.98	All	NA	0.99 (0.49‐2.01)

*Note*: Values of *P* < .05 were considered significant.

Abbreviations: ALP, alkaline phosphatase; ALT, alanine aminotransferase; Bands, band neutrophils; BUN, blood urea nitrogen; Eos, eosinophils; GBM type, gallbladder mucocele ultrasonographic classification; HCT, hematocrit; Lymphs, lymphocytes; Med, medically managed group; Med‐Sx, Medical then surgical group; Monos, monocytes; Segs, segmented neutrophils; Sx, surgical treatment group; WBC, white blood cells.

a *P*‐values calculated from univariate Cox model.

b *P*‐values and hazard ratios calculated for each group from multivariate Cox model when interaction between group, variable, and survival was suspected (*P* < .15).

c Hazard ratio unit = 100.

*P < .05 and a hazard ratio that did not include 1.0.

Statistical analysis was performed using commercially available statistical software (SAS V 9.3, Cary, North Carolina) and significance was set at *P* < .05.

## 3 RESULTS

### 3.1 Animals

Eighty‐nine dogs (3 [3.3%] intact females, 3 [3.3%] intact males, 41 [46%] spayed females, and 42 [47.2%] castrated males) with a mean age of 10.8 years (SD, 3.2 years) were included in the study (Table 2). Patients' age and sex were not significantly associated with survival. Dog breeds included mixed breed (n = 8), Cocker Spaniels (7), Shetland Sheepdogs (6), Bichon Frises (6), Jack Russell Terriers (6), West Highland White Terriers (5), Yorkshire Terriers (5), Pomeranians (4), Labrador Retrievers (4), Miniature Schnauzers (4), Miniature Poodles (4), Pugs (4), Beagles (2), Collies (2), Golden Retrievers (2), Lhasa Apsos (2), Maltese (2), and (16) various other breeds represented by 1 dog each.

**Table 2:** Patient sex, neuter status, and age by treatment group

	Sx # (%)	Med # (%)	Med‐Sx # (%)
FI	2 (4)	1 (3)	0 (0)
MI	3 (7)	0 (0)	0 (0)
FS	15 (33)	21 (64)	5 (50)
MC	26 (57)	11 (33)	5 (50)
Total	46	33	10
Age^a^ (years)	10.7 ± 2.7	10.7 ± 3.8	9.7 ± 2.8

*Notes*: Sex and neuter status reported as #, total number; (%), percent within treatment group. Age reported as mean age in years ±SD within treatment group.

Abbreviations: FI, female intact; FS, female spayed; MC, male castrated; Med, medically managed group; Med‐Sx, medical then surgical group; MI, male intact; Sx, surgical treatment group.

a Mean within treatment group.

There were 46 (51.6%) dogs in the surgical treatment group (Sx), 33 (37.1%) dogs in the medical treatment group (Med), and 10 (11.2%) dogs that initially underwent medical management with subsequent surgery (Med‐Sx). Patient breed (*P* = .22), age (*P* = .32), and GBM type (*P* = .19) were not significantly different between dogs in the medically managed group and dogs in the surgical group. There were significantly more spayed females (60.5 versus 32.6%) and fewer neutered males (37.2 versus 56.5%) in the medically managed group versus the surgical group, respectively (*P* = .02).

### 3.2 Clinicopathologic results

Sixty‐nine (77.5%) dogs had data available for a CBC whereas 84 (93.3%) dogs had data available for serum biochemistry. Biochemical abnormalities included increased alkaline phosphatase (ALP) activity (n = 70 of 77 [90.9%]), increased alanine aminotransferase activity (57 of 77 [74.0%]), hyperbilirubinemia (39 of 75 [52.0%]), hypoalbuminemia (36 of 75 [48%]), hypercholesterolemia (35 of 74 [47.3%]), ionized hypocalcemia (13 of 28 [46.4%]), hypoproteinemia (18 of 72 [25.0%]), hyponatremia (18 of 74 [24.3%]), hypochloremia (16 of 71 [22.5%]), hyperchloremia (15 of 71 [21.1%]), total hypocalcemia (12 of 64 [18.8%]), increased blood urea nitrogen concentration (14 of 81 [17.3%]), hypernatremia (12 of 74 [16.2%]), hyperglycemia (13 of 82 [15.9%]), hypoglycemia (13 of 82 [15.9%]), hypermagnesemia (9 of 58 [15.5%]), total hypercalcemia (9 of 64 [14.1%]), hypokalemia (10 of 74 [13.5%]), increased serum creatinine concentration (9 of 73 [12.3%]), hyperkalemia (9 of 74 [12.2%]), hyperphosphatemia (7 of 64 [10.9%]), hypomagnesemia (6 of 58 [10.3%]), low serum bicarbonate concentration (6 of 69 [8.7%]), hyperproteinemia (6 of 72 [8.3%]), hypophosphatemia (4 of 64 [6.3%]), high serum bicarbonate concentration (4 of 69 [5.8%]), hypocholesterolemia (3 of 74 [4.1%]), ionized hypercalcemia (1 of 26 [3.6%]), and increased serum albumin concentration (2 of 75 [2.7%]). Complete blood count abnormalities included mature neutrophilia (46 of 68 [67.7%]), leukocytosis (37 of 69 [53.6%]), anemia (30 of 70 [42.9%]), monocytosis (24 of 68 [35.3%]), thrombocytosis (21 of 70 [30.0%]), thrombocytopenia (19 of 70 [27.1%]), left shift (15 of 64 [23.4%]), lymphopenia (9 of 68 [13.2%]), monocytopenia (3 of 68 [4.4%]), basophilia (2 of 67 [3.0%]), lymphocytosis (1 of 68 [1.5%]), and eosinophilia (1 of 67 [1.5%]) (Table 3).

**Table 3:** Clinicopathologic descriptive data by treatment group

		Sx			Med			Med‐Sx		
Variable	Reference	Mean ± SD	# (%) HI	# (%) LO	Mean ± SD	# (%) HI	# (%) LO	Mean ± SD	# (%) HI	# (%) LO
WBC (×10^3^ cells/μL)	4.2‐12.9	18.8 ± 10.8	20 (62.5)	0 (0)	13.9 ± 8.5	12 (41.4)	0 (0)	15.7 ± 6.96	5 (56)	0 (0)
Hct (%)	42.2‐59.8	42.5 ± 6.3	0 (0)	15 (45.5)	41.8 ± 8.5	0 (0)	11 (37.9)	42.3 ± 7	0 (0)	4 (44)
Segs (×10^3^ cells/μL)	2.7‐8.5	16.2 ± 9.4	26 (78.8)	0 (0)	11.3 ± 8	13 (48.1)	0 (0)	13.1 ± 6.6	7 (78)	0 (0)
Bands (×10^3^ cells/μL)	0‐0.3	0.39 ± 0.78	11 (33.3)	0 (0)	0.3 ± 0.8	4 (16.0)	0 (0)	0 ± 0	0 (0)	0 (0)
Lymphs (×10^3^ cells/μL)	0.5‐4.1	1.1 ± 0.78	1 (3.0)	5 (15.2)	1.3 ± 0.8	0 (0)	3 (11.1)	1.18 ± 0.7	0 (0)	1 (11)
Monos (×10^3^ cells/μL)	0.1‐1	1.51 ± 1.46	15 (42.4)	0 (0)	0.7 ± 0.8	5 (18.5)	3 (11.1)	1.21 ± 0.7	5 (56)	0 (0)
Eos (×10^3^ cells/μL)	0‐1.2	0.21 ± 0.41	1 (3.0)	0 (0)	0.2 ± 0.3	0 (0)	0 (0)	0.28 ± 0.13	0 (0)	0 (0)
Platelets (×10^3^ cells/μL)	226‐424	302 ± 152	8 (24.2)	12 (36.4)	360 ± 166	9 (31.0)	6 (20.7)	405 ± 139	5 (56)	1 (11)
Sodium (mmol/L)	143‐150	145 ± 4.8	3 (7.7)	11 (28.2)	145.6 ± 5.8	7 (24.1)	7 (24.1)	146.7 ± 5.4	2 (25)	1 (12.5)
Potassium (mmol/L)	3.5‐5.1	4.1 ± 0.6	2 (5.1)	6 (15.4)	4.5 ± 0.7	7 (24.1)	4 (13.8)	4.6 ± 0.3	1 (12.5)	0 (0)
Chloride (mmol/L)	106‐114	110 ± 6.3	8 (21.1)	8 (21.1)	108.9 ± 7.6	6 (22.2)	7 (25.9)	96.9 ± 35.1	1 (12.5)	2 (25)
Bicarbonate (mmol/L)	16‐25	20.2 ± 3.6	3 (8.1)	3 (8.1)	20.1 ± 3.7	1 (3.7)	3 (11.1)	20.4 ± 2.9	0 (0)	0 (0)
BUN (mg/dL)	9‐28	15.5 ± 12.5	3 (7.0)	9 (20.9)	25.6 ± 19.3	10 (32.3)	3 (9.8)	11.2 ± 10.7	1 (12.5)	5 (62.5)
Creatinine (mg/dL)	0.6‐1.3	0.96 ± 0.8	5 (13.2)	7 (18.4)	1.01 ± 0.5	4 (13.8)	3 (10.3)	0.76 ± 0.25	0 (0)	1 (12.5)
Total protein (g/dL)	5.4‐7.1	5.8 ± 1.2	3 (8.6)	13 (37.1)	6.1 ± 0.7	2 (6.5)	6 (19.4)	6.4 ± 0.7	1 (12.5)	0 (0)
Albumin (g/dL)	3.3‐4.2	3.1 ± 0.7	1 (2.8)	18 (50.0)	3.3 ± 0.6	1 (3.1)	14 (43.8)	3.0 ± 0.7	0 (0)	6 (67)
ALP (×10^3^ U/L)^a^	13‐95	2.8 ± 3.0	35 (92.1)	0 (0)	1.5 ± 2.2	28 (87.5)	0 (0)	2.6 ± 3.6	9 (100)	0 (0)
ALT (U/L)	12‐106	848 ± 898	33 (86.8)	0 (0)	377 ± 496	18 (56.3)	0 (0)	479 ± 398	8 (89)	0 (0)
Glucose (mg/dL)	76‐120	91 ± 29	5 (11.6)	11 (25.6)	106 ± 35	5 (16.1)	2 (6.5)	137.4 ± 77	3 (30)	0 (0)
Calcium, ionized (mmol/L)	1.2‐1.5	1.2 ± 0.2	1 (5.9)	8 (47.1)	1.2 ± 0.14	0 (0)	5 (55.6)	1.23 ± .001	0 (0)	0 (0)
Calcium, total (mg/dL)	9.5‐11.2	10.1 ± 1.2	4 (13.3)	8 (26.7)	10.3 ± 1.0	4 (13.8)	4 (13.8)	10.4 ± 1	1 (14)	1 (14)
Phosphorus (mg/dL)	2.7‐5.2	4.2 ± 2.2	3 (10.0)	2 (6.7)	4.5 ± 1.7	4 (13.8)	1 (3.4)	3.96 ± 0.8	0 (0)	1 (14)
Magnesium (mg/dL)	1.8‐2.4	2.1 ± 0.4	4 (13.8)	5 (17.2)	2.3 ± 0.3	4 (16.0)	1 (3.4)	2.14 ± 0.3	1 (20)	0 (0)
Cholesterol (mg/dL)	109‐345	378 ± 180	19 (51.4)	1 (2.7)	393 ± 275	14 (45.2)	1 (3.2)	351 ± 336	3 (37.5)	1 (12.5)
Bilirubin (mg/dL)	0‐0.3	4.7 ± 6.4	26 (68.4)	0 (0)	1.4 ± 2.2	10 (33.3)	0 (0)	1.6 ± 2.9	4 (44)	0 (0)

Abbreviations: # (%) HI, number of dogs (% within treatment group) above the reference range; # (%) LO, number of dogs (% within treatment group) below the reference range; ALP, alkaline phosphatase; ALT, alanine aminotransferase; Bands, band neutrophils; BUN, blood urea nitrogen; Eos, eosinophils; HCT, hematocrit; Lymphs, lymphocytes; Med, medically managed group; Med‐Sx, medical then surgical group; Monos, monocytes; Segs, segmented neutrophils; Sx, surgical treatment group; WBC, white blood cells.

a ALP activity is presented as 10^3^ U/L.

In the 3‐group model, dogs with increased serum creatinine concentration, hyperphosphatemia, and increased ALP activity were significantly more likely to have a shorter survival time (Table 1). Serum creatinine and phosphorus concentrations were found to be significant for survival across all treatment groups in the univariate model but had no effect in the multivariate model. Serum ALP activity was found to be significantly associated with outcome across all treatment groups in the univariate analysis, but HRs calculated with the multivariate model indicated that patients with increased ALP activity in the surgical group to be at highest risk of mortality. No other clinicopathologic variables evaluated were significant for survival, including serum total bilirubin concentration.

### 3.3 Abdominal ultrasonographic findings

Ultrasonographic appearance of the gallbladder (GBM type) was significantly different among treatment groups with 77 (86.5%) dogs having ultrasound images available for review (Table 4). Nine of 77 (11.7%) dogs were characterized as type 1, 31 (40.2%) as type 2, 21 (27.3%) as type 3, 7 (9%) as type 4, 7 (9%) as type 5, and 0 as type 6. In the 3‐group model, a higher GBM type was significantly associated with decreased survival in all treatment groups (*P* = .05; HR, 1.41; 95% CI, 1.01‐1.98).

**Table 4:** Ultrasound classification of biliary mucoceles (GBM type)

GBM type	Description	Sx # (%)	Med # (%)	Med‐Sx # (%)
1	Immobile echogenic bile	4 (11)	4 (13)	1 (12.5)
2	Incomplete stellate pattern	12 (32)	16 (52)	3 (38)
3	Typical stellate pattern	10 (26)	9 (29)	2 (25)
4	Kiwi‐like pattern and stellate combination	4 (11)	2 (6)	1 (12.5)
5	Kiwi‐like pattern with residual central echogenic bile	6 (16)	0 (0)	1 (12.5)
6	Kiwi‐like pattern	0 (0)	0 (0)	0 (0)
Rupture	Concern for GB rupture in the ultrasound report	18 (47)	3 (10)	2 (25)

Abbreviations: # (%), number of dogs (% within treatment group) with associated GBM type; GB, gallbladder; GBM type, gallbladder mucocele ultrasonographic classification; Med, medically managed group; Med‐Sx, medical then surgical group; Sx, surgical treatment group.

Biliary rupture was suspected in 23 of 77 (29.9%) dogs based on ultrasound imaging findings, the presence of which was significantly associated with improved survival in surgical patients in the 3‐group model (*P* = .02; HR, 0.21; 95% CI, 0.06‐0.76). Suspicion of biliary rupture did not significantly impact outcome in the Med (HR, 0.86; 95% CI, 0.11‐6.9) and Med‐Sx (HR, 1.66; 95% CI, 0.33‐8.38) groups. For dogs undergoing surgery (Sx and Med‐Sx), 20 of 46 dogs (43.5%) with available ultrasound imaging were reported to have changes consistent with or suspicious of gall bladder rupture preoperatively, and 7 of these 20 (35.0%) were confirmed to have biliary rupture during surgery.

### 3.4 Surgical versus medical management

**Figure 1:**
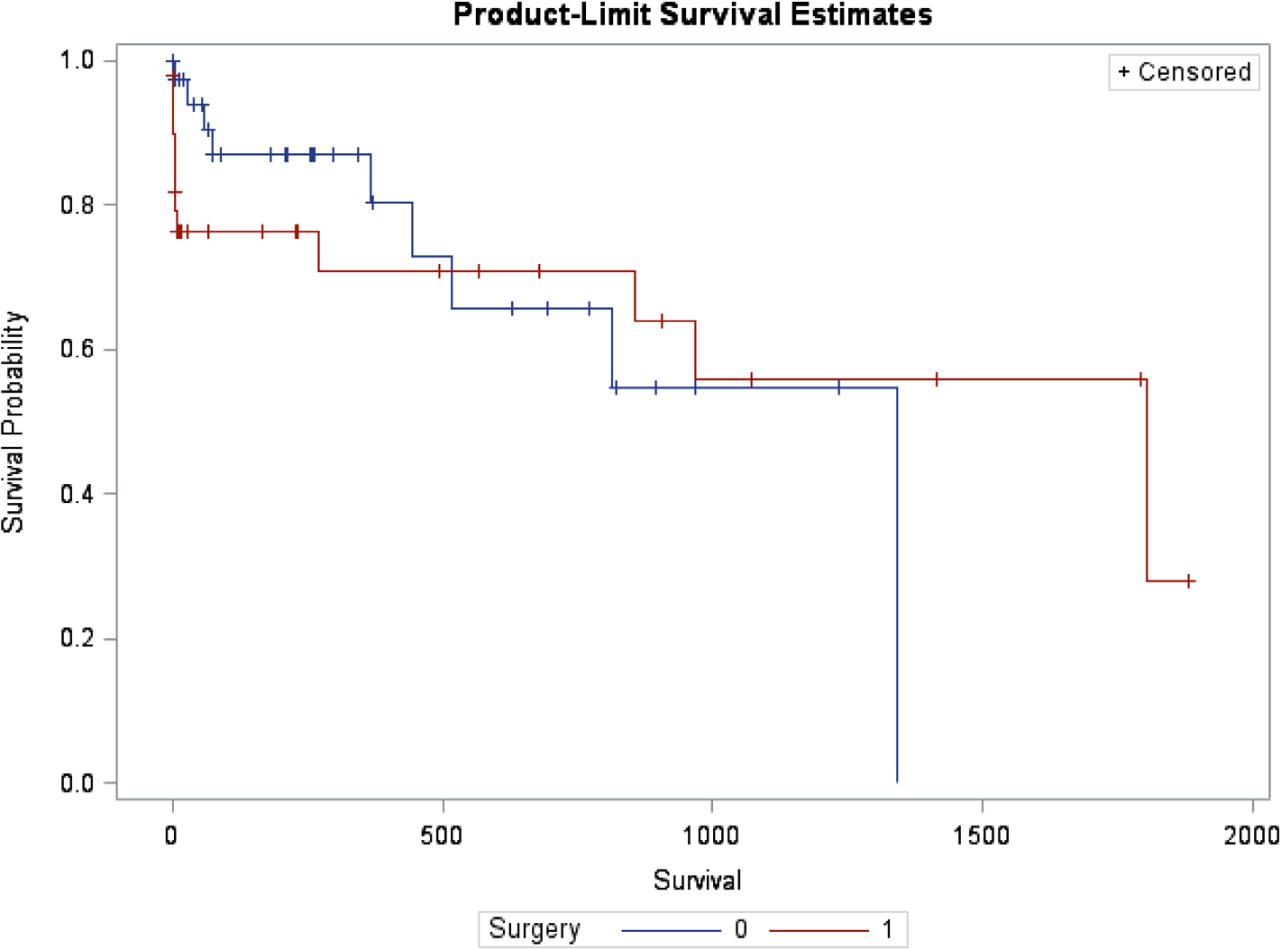
Survival probability based on 2‐group model: Sx group (surgery = 1, dogs primarily treated with cholecystectomy) versus Med and Med‐Sx groups (surgery = 0, dogs that were primarily treated medically). The survival probability crosses at approximately 500 days postdiagnosis therefore failing to meet the assumption of proportional hazards

Survival curves for medical management versus surgical management using proportional hazards analysis crossed at approximately 500 days (Figure 1). Therefore, the assumption of proportional hazards was not met. The 14‐day mortality rates of the Sx, Med, and Med‐Sx groups were 19.6, 3.0, and 0%, respectively, which does not include censored individuals. The number of dogs lost to follow‐up in the first 14 days after diagnosis in the Sx, Med, and Med‐Sx groups were 12 of 46 (26.1%), 6 of 33 (18.2%), and 0 of 10 (0%), respectively. The median follow‐up duration was 39 (range, 0‐1823) days for all dogs, 13.5 (range, 0‐1823) days in the Sx group, 210 (range, 0‐1340) days in the Med group, and 123.5 (range, 22‐1150) days in the Med‐Sx group. In dogs that were treated medically before surgery (Med‐Sx), the mean duration of medical management before surgery was 90.6 ± 124.5 days.

Cholecystocentesis and bile cytology was performed in 12 dogs. Cytology identified bactibilia in 4 dogs, inspissated and mucinous bile in 4 dogs, cytologically normal bile in 3 dogs, and cystic fluid in 1 dog. Results of bacterial culture of bile, gallbladder tissue, or both were available in 24 dogs. Four dogs had positive culture results with *Enterococcus* sp., *Bacillus* sp., *Clostridium* sp., and *Lactobacillus* sp. identified once each. The remaining 20 bile samples had no bacterial growth or isolated contaminant bacteria. Dogs in all groups received a variety of medical treatments including UDCA, cytoprotective agents or supplements such as S‐adenosyl methionine (SAMe), opioid pain medications, antibiotics, and a variety of other medications in fewer numbers (see Supplemental Table S1). A total of 86 antibiotic courses were prescribed in 56 dogs from all treatment groups. Fifty‐four of these dogs were treated empirically, at the discretion of the attending veterinarian, without a positive culture result. The 2 dogs with positive bile culture results that were not prescribed antibiotics were both treated surgically and discharged from the hospital before culture results were available. Follow‐up information is not available for either of the dogs.

Of the dogs that were treated medically (Med group), 30 of 33 (90.9%) dogs received UDCA at dosages ranging from 4.8 to 20 mg/kg and dose frequency ranging from q12h to q24h, and 20 of 33 (60.6%) dogs received SAMe (dosage rarely reported). Other treatments in the medically managed group included maropitant (n = 6), proton pump inhibitors (n = 6), opioids (n = 6), amoxicillin clavulanate (n = 6), enrofloxacin (n = 4), metronidazole (n = 4), glucocorticoids (n = 4), histamine receptor antagonists (n = 4), serotonin reuptake inhibitors (n = 4), vitamin E (n = 4), trilostane (n = 4), and angiotensin converting enzyme inhibitors (n = 4). A variety of other medications were administered to patients in fewer numbers (Supplemental Table S1).

**Figure 2:**
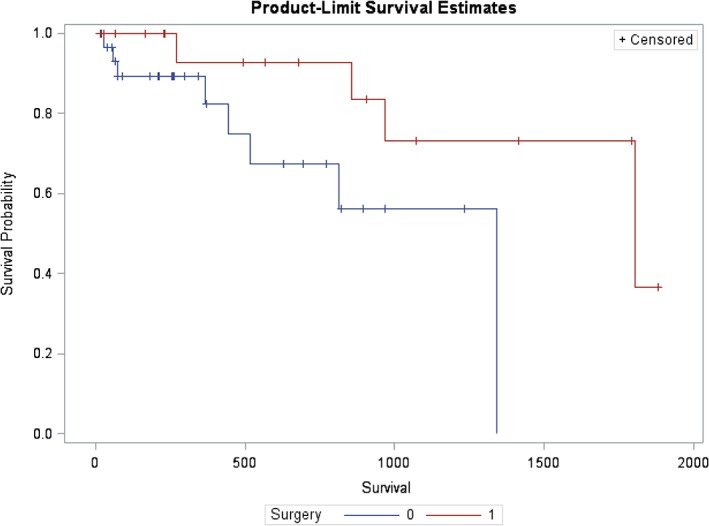
Survival probability based on the 2‐group model starting 14 days postdiagnosis with a gallbladder mucocele (GBM). Dogs initially treated with cholecystectomy (surgery = 1) demonstrate a better long‐term survival compared to dogs initially treated medically (surgery = 0); *P* = .04, hazard ratio, 4.13 (1.02‐16.82)

**Figure 3:**
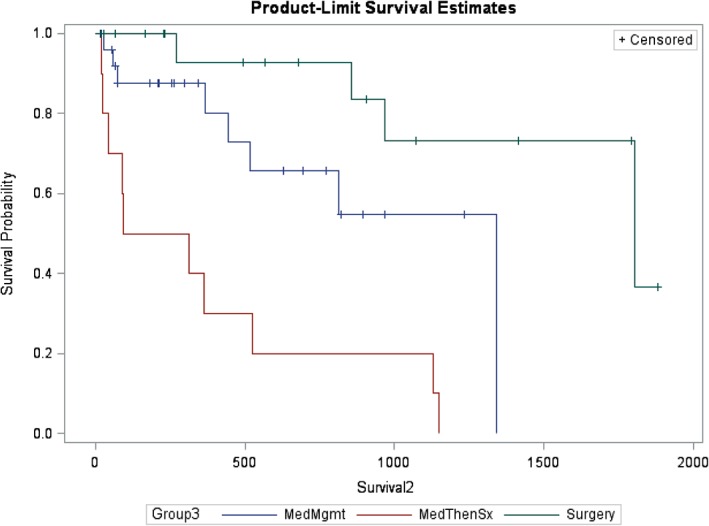
Survival probability based on the 3‐group model starting 14 days postdiagnosis with a gallbladder mucocele (GBM). Sx group, surgery (median survival time [MST] 1802 days [855‐not reached]); Med group, MedMgmt (MST 1340 days [444‐1340]); Med‐Sx group, MedThenSx (MST 203 days (18‐525). Survival probabilities were significantly different between groups (*P* < .0001) with a 4 times greater risk of death in the Med group (hazard ratio [HR], 3.99 [1.007‐15.82]) and 14 times greater risk of death in the Med‐Sx group (HR, 14.0 [3.6‐54.9])

Survival analysis of dogs in the medically managed groups (Med and Med‐Sx) compared to the surgical group, starting at 14 days after initial diagnosis to minimize the effect of initial treatment decisions such as euthanasia and perioperative mortality (Figure 2), showed significantly shorter survival (*P* = .04; HR, 4.13; 95% CI, 1.02‐16.82) in the medically managed group (median survival time [MST], 1340 days; 95% CI, 444‐1340) versus the surgical group (MST, 1802 days [range, 855‐not reached]). When separated into 3 groups (Sx, Med, and Med‐Sx), the Med group had significantly shorter survival (MST, 1340 days; 95% CI, 444‐1340; *P* < .0001; HR, 3.99; 95% CI, 1.007‐15.82) compared to the Sx group (MST, 1802 days; range, 855‐not reached). The Med‐Sx group had a MST of 203 days (range, 18‐525) with a 14 times greater chance of death compared to the Sx group (HR, 14.0; 95% CI, 3.6‐54.9; Figure 3).

## 4 DISCUSSION

Cholecystectomy is commonly performed in small animal practice and is regarded as the standard of care treatment for GBM but has historically been associated with high mortality rates (22%‐40%).^1^, ^2^, ^3^, ^4^, ^5^, ^6^, ^7^, ^8^ Results of our study suggest a 14‐day mortality rate of 19.6%, similar to previous reports of dogs undergoing cholecystectomy. Most studies have evaluated cholecystectomy in patients presenting on emergency or ill, although it is generally accepted that incidentally diagnosed mucoceles or early mucoceles are being diagnosed with increasing frequency. The cause of the increased incidence is not known but is hypothesized to be associated with increased routine use of abdominal ultrasonography and increased vigilance for biliary disease. Recommending cholecystectomy for incidentally diagnosed GBM has been controversial because, until recently, no studies had evaluated surgical outcomes in this population of patients. A recent study found significantly lower mortality (2%) in dogs undergoing elective cholecystectomy compared to those undergoing emergency cholecystectomy (22%‐40%).^9^ Some clients still are reluctant to pursue surgical intervention for a variety of reasons but no studies to date have evaluated the outcomes of medical management for GBM. In our study, surgical treatment of GBM resulted in a significantly longer MST after initial diagnosis compared to dogs treated with medical management alone or with medical management followed by surgical treatment. Survival analysis excluded patients that were censored or died in the first 14 days after treatment so as to compare long‐term outcomes with surgical and medical treatment options, which may have skewed overall survival times to be longer. Better long‐term outcome in the surgical group compared to the medically managed group corroborates that cholecystectomy may be the ideal treatment for dogs with GBM, but medical management can provide reasonable outcomes if surgery is declined. The cause of decreased survival in the Med‐Sx group relative to the other groups is unclear. No variables were identified that predicted failure of medical treatment or decreased survival in that group relative to the others. Fewer patients in the Med‐Sx group as well as the retrospective nature of the study may have prevented us from identifying prognostic factors that would have predicted medical failure. Future prospective studies are needed to identify which patients would benefit the most from early surgical intervention.

Consistent with previous studies of GBM in dogs, Cocker Spaniels, Shetland Sheepdogs, and Miniature Schnauzers were well represented in our study,^1^, ^5^, ^6^, ^11^, ^12^ although mixed breed dogs were most commonly affected, which is not as commonly reported. Sex and neuter status were significantly different among groups, but there was no association with outcome in any treatment group suggesting that sex and neuter status did not impact survival.

In our study, increased serum creatinine concentration and hyperphosphatemia were associated with decreased survival regardless of treatment group. This finding is similar to that of previous reports of negative prognostic factors associated with biliary surgery in humans and dogs.^28^, ^29^, ^30^ The cause of decreased survival associated with decreased renal function is likely multifactorial. Chronic kidney disease alone negatively impacts long‐term prognosis, but also has implications for renal handling of toxins and medications.^31^ Acute kidney injury occurring secondary to primary disease, such as pigment nephropathy, as can occur because of bilirubinuria, also is possible and may affect short‐ and long‐term prognosis.^32^ In our study, it is unknown which measures were undertaken for each patient to stabilize and correct fluid deficits before initiation of treatment, which may have contributed to the significance of the serum creatinine and phosphorus concentrations. Additionally, our study identified increased ALP activity as a negative prognostic factor across treatment groups but most significantly in the surgical group, which is similar to findings in humans undergoing biliary surgery and is suspected to be related to the severity of cholestatic disease.^29^ Increased serum total bilirubin concentration and white blood cell count were associated with negative outcomes in previous studies, which was not found in our study.^4^

Abdominal ultrasound examination remains the most common method to diagnose GBM, and several descriptions and scoring systems have been described.^12^ Detection of “early” or “pre‐” mucoceles may be enhanced by the presence of ultrasonographically scored gallbladder debris and potentially may allow for prognostication based on severity of GBM. In our study, increasing severity of GBM type was significantly associated with decreased survival in all groups, suggesting that ultrasound images may allow better prognostication beginning treatment. This finding also suggests that early detection of low‐scoring GBM may allow for earlier intervention and improved outcomes. The presence of biliary rupture inconsistently has been associated with poor outcome in dogs undergoing biliary surgery, with some reports suggesting no association with survival^1^, ^4^ and others reporting decreased survival.^33^ In our study, suspicion of biliary rupture was based on the presence of effusion in the gallbladder fossa, pericholecystic echogenic fat, and presence of discontinuous gallbladder wall or some combination of these findings. Suspicion of biliary rupture was associated with improved survival in the surgical group compared to dogs without suspicion of biliary rupture on ultrasound examination, which is contrary to previous studies.^1^, ^4^, ^33^ The cause of this discrepancy is unknown, but increased vigilance for impending or early rupture may prompt more rapid surgical intervention and result in improved outcomes. This possibility is further supported by the fact that AUS examination overestimated the presence of biliary rupture in our study (43.5 versus 18.9%), which may have prompted a more aggressive approach to treatment by the attending clinician. Further investigation into this possibility is warranted.

Limitations of our study include its retrospective nature in which diagnostic approach, recommended treatments, and case follow‐up varied by the attending clinician. Several patients initially were diagnosed with GBM at nearby referring hospitals and GBM was not always confirmed by in‐house ultrasonography, which may have resulted in further variation in diagnostic criteria for GBM in our study. Many cases were censored because of lack of follow‐up data which may have skewed our survival data. Future prospective studies should assess the efficacy of medical treatment for GBM to allow for a standardized medical approach and monitoring for resolution or progression.

In summary, our study showed that treatment by cholecystectomy is associated with the best long‐term outcome in patients with GBM, particularly if diagnosed early. Medical management is a feasible option with a MST of 1340 days for clients who decline cholecystectomy. Although serum creatinine and phosphorus concentrations, ALP activity, and GBM scores were found to be prognostic indicators in our study, prospective studies directly comparing standardized approaches to both surgically and medically treated dogs with GBM are needed.

## CONFLICT OF INTEREST DECLARATION

Authors declare no conflict of interest.

## OFF‐LABEL ANTIMICROBIAL DECLARATION

Authors declare no off‐label use of antimicrobials.

## INSTITUTIONAL ANIMAL CARE AND USE COMMITTEE (IACUC) OR OTHER APPROVAL DECLARATION

Authors declare no IACUC or other approval was needed.

## HUMAN ETHICS APPROVAL DECLARATION

Authors declare human ethics approval was not needed for this study.

## Supporting information


**Supplemental Table S1** Medical treatments administered to patients associated with treatment of GBM or historical diagnoses
