# Supplementary material for: Long‐term survival of dogs treated for gallbladder mucocele by cholecystectomy, medical management, or both
Source: J Vet Intern Med. 2019 Sep 6;33(5):2057–66. doi: 10.1111/jvim.15611 (PMC6766501; doi:10.1111/jvim.15611)
Supplement: Supplementary file 1 — Supplemental Table S1 Medical treatments administered to patients associated with treatment of GBM or historical diagnoses [file JVIM-33-2057-s001.pdf]

Supplemental Table 1 – Medical treatments administered to patients associated with treatment of GBM or historical diagnoses.

Medication	All dogs # (%)	Sx # (%)	Med # (%)	Med-Sx # (%)
Ursodeoxycholic acid (UDCA)	61 (68.5)	22 (47.8)	30 (90.9)	9 (90)
Opioid	45 (50.6)	35 (76.1)	6 (18.2)	4 (40)
S-adenosyl methionine (SAMe)	42 (47.2)	20 (43.5)	16 (48.5)	6 (60)
Enrofloxacin	24 (26.9)	14 (30.4)	4 (12.1)	6 (6)
Amoxicillin clavulanate	23 (25.8)	15 (32.6)	6 (18.2)	2 (20)
Proton pump inhibitor	19 (21.3)	12 (26.1)	6 (18.2)	1 (10)
Histamine H2 receptor antagonist	17 (19.1)	11 (23.9)	4 (12.1)	2 (20)
Maropitant	16 (17.9)	8 (17.4)	6 (18.2)	2 (20)
Metronidazole	11 (12.4)	6 (13.0)	4 (12.1)	1 (10)
Serotonin reuptake inhibitors	11 (12.4)	5 (10.9)	4 (12.1)	2 (20)
Glucocorticoids	10 (11.2)	4 (8.7)	4 (12.1)	2 (20)
Levothyroxine	10 (11.2)	7 (15.2)	2 (6.1)	1 (10)
ACEi	9 (10.1)	4 (8.7)	4 (12.1)	1 (10)
Metoclopramide	8 (8.9)	6 (13.0)	1 (3.0)	1 (10)
Ampicillin	7 (7.9)	2 (4.4)	0 (0)	5 (50)
Amoxicillin	7 (7.9)	2 (4.4)	3 (9.1)	2 (20)
Vitamin E	7 (7.9)	1 (2.2)	4 (12.1)	2 (20)
Ampicillin Sulbactam	6 (6.7)	6 (13.0)	0 (0)	0 (0)
N-acetylcysteine	6 (6.7)	5 (10.8)	1 (3.0)	0 (0)
Trilostane	6 (6.7)	1 (2.2)	4 (12.1)	1 (10)
Insulin	6 (6.7)	1 (2.2)	4 (9.1)	2 (20)
Amlodipine	5 (5.6)	3 (6.5)	1 (3.0)	0 (0)
Phenobarbital	4 (4.5)	3 (6.5)	1 (3.0)	0 (0)
Mirtazapine	4 (4.5)	2 (4.4)	2 (6.1)	0 (0)
NSAID (systemic)	4 (4.5)	3 (6.5)	1 (3.0)	0 (0)
Vitamin K	4 (4.5)	4 (8.7)	0 (0)	0 (0)

Medication	All dogs # (%)	Sx # (%)	Med # (%)	Med-Sx # (%)
Cyclosporine (ophthalmic)	3 (3.4)	1 (2.2)	2 (6.1)	0 (0)
Omega-3 fatty acid	3 (3.4)	0 (0)	3 (9.1)	0 (0)
Sucralfate	3 (3.4)	0 (0)	3 (9.1)	0 (0)
NSAID (ophthalmic)	3 (3.4)	0 (0)	3 (9.1)	0 (0)
Ciprofloxacin	2 (2.3)	1 (2.2)	1 (3.0)	0 (0)
Ticarcillin Clavulanate	2 (2.3)	2 (4.4)	0 (0)	0 (0)
Furosemide	2 (2.3)	2 (4.4)	0 (0)	0 (0)
Fibrates	2 (2.3)	0 (0)	2 (6.1)	0 (0)
Glutamine	2 (2.3)	2 (4.4)	0 (0)	0 (0)
Phenylpropanolamine	2 (2.3)	2 (4.4)	0 (0)	0 (0)
Trazodone	2 (2.3)	1 (2.2)	0 (0)	1 (10)
Diphenhydramine	2 (2.3)	1 (2.2)	1 (3.0)	0 (0)
Cefpodoxime	1 (1.1)	0 (0)	1 (3.0)	0 (0)
Cephalexin	1 (1.1)	0 (0)	1 (3.0)	0 (0)
Doxycycline	1 (1.1)	0 (0)	0 (0)	1 (10)
Clindamycin	1 (1.1)	0 (0)	1 (3.0)	0 (0)
Levetiracetam	1 (1.1)	1 (2.2)	0 (0)	0 (0)
Plasma transfusion	1 (1.1)	1 (2.2)	0 (0)	0 (0)
Amitriptyline	1 (1.1)	1 (2.2)	0 (0)	0 (0)
Theophylline	1 (1.1)	0 (0)	1 (3.0)	0 (0)
Pimobendan	1 (1.1)	1 (2.2)	0 (0)	0 (0)
Norepinephrine	1 (1.1)	1 (2.2)	0 (0)	0 (0)
Fenbendazole	1 (1.1)	0 (0)	1 (3.0)	0 (0)
Spironolactone	1 (1.1)	0 (0)	1 (3.0)	0 (0)
Vinblastine	1 (1.1)	0 (0)	1 (3.0)	0 (0)
Aluminum hydroxide	1 (1.1)	1 (2.2)	0 (0)	0 (0)
Mycophenolate	1 (1.1)	0 (0)	1 (3.0)	0 (0)
Clopidogrel	1 (1.1)	0 (0)	1 (3.0)	0 (0)

Medication	All dogs # (%)	Sx # (%)	Med # (%)	Med-Sx # (%)
Cobalamine	1 (1.1)	0 (0)	0 (0)	1 (10)
Misoprostol	1 (1.1)	1 (2.2)	0 (0)	0 (0)
Gabapentin	1 (1.1)	0 (0)	1 (3.0)	0 (0)
Barium	1 (1.1)	0 (0)	1 (3.0)	0 (0)
Lidocaine	1 (1.1)	0 (0)	1 (3.0)	0 (0)

Sx, surgical treatment group; Med, medically managed group; Med-Sx, medical then surgical group; ACEi, angiotensin converting enzyme inhibitor; NSAID, non-steroidal anti-inflammatory drug.

Number of dogs receiving a medication reported as #, total number within treatment group; and (%), percent within treatment group.
